# The cross-sectional relationship of physical frailty and cognitive impairment in older Poles: analysis of the PolSenior2 data

**DOI:** 10.1007/s41999-025-01220-0

**Published:** 2025-05-17

**Authors:** Anna Skalska, Karolina Piotrowicz, Hanna Kujawska-Danecka, Kacper Jagiełło, Alicja Klich Rączka, Małgorzata Mossakowska, Tomasz Zdrojewski, Tomasz Grodzicki, Jerzy Gąsowski

**Affiliations:** 1 Department of Internal Medicine and Gerontology, Jagiellonian University Medical College, Kraków, Poland; 2 Department of Rheumatology, Clinical Immunology, Geriatrics and Internal Medicine, Medical Univeristy of Gdansk, Gdańsk, Poland; 3 Department of Preventive Medicine and Education, Medical University of Gdansk, Gdańsk, Poland; 4 Study on Aging and Longevity, International Institute of Molecular and Cell Biology, Warsaw, Poland

**Keywords:** Physical frailty, Cognitive impairment, Frailty components, Older adults

## Abstract

**Aim:**

To assess the relation between pre-frailty and frailty, and its components on one hand and the presence, degree and components of cognitive impairment.

**Methods:**

This is a nationwide epidemiologic survey of health determinants in older Poles (PolSenior2). We assessed physical frailty using the approach by Fried et al. and cognitive impairment with the Mini-Mental Status Examination (MMSE). We calculated population estimates (95% CI) of conditions under study, with sampling weights to account for the complex survey design, with the age–sex post-stratification matching to the population of Poland. We used logistic regression to model cognitive impairment as function of frailty phenotype.

**Results:**

Mean (SD) age of 5378 participants (58.0% women) was 75.0 (9.4) years. Frailty was diagnosed in 23.5% of respondents, and 54.6% were pre-frail. Normal cognition was found in 51.3%, 23.0% had suspected mild cognitive impairment (MCI), and suspected: 16.3% mild, 6.5% moderate and 2.9% severe dementia. Robust phenotype was present in 35.7% normal cognition persons, 4.7% in moderate dementia, and 0.0% in severe dementia. Frailty increased from 9.2% in normal cognition to 57.4% and 85.3% in moderate dementia and severe dementia. Frailty component that was associated with abnormalities in all MMSE domains was exhaustion. In the presence of exhaustion, the best-preserved function was registration, and the most pronounced was attention deficit.

**Conclusion:**

Frailty and cognitive impairment are closely associated. While exhaustion was standing out among the frailty components, it was associated with impaired orientation and attention deficits. These data may inform both the prevention and rehabilitation and nutritional counseling.

## Key summary points

**Aim:**

To assess the relation between physical frailty and cognitive impairment, their components and stage.

**Findings:**

Physical frailty is associated with more pronounced cognitive decline. Of frailty components, exhaustion associates with deficits in attention, orientation and to some extent short-term memory and visuo-spatial abilities.

**Message:**

Our results may inform the preventive and therapeutic approaches, especially rehabilitation.

## Introduction

Frailty is linked to increased morbidity and mortality [1]. It is also a potent modifier of risk related to other disease states, ranging from hypertension to urgent surgery [2, 3]. This is a consequence of an interplay between inflammaging, mitochondrial dysfunction and impaired cellular regulation [4]. The screening for physical frailty has been specifically recommended in older oncological, hematological, surgical and cardiovascular patients, at all stages of disease and its treatment [1]. It has been demonstrated that the diagnosis of physical frailty meaningfully influenced therapeutic decisions [3, 5]. In patients preparing for such demanding treatment modalities as chemotherapy or surgery, the diagnosis of physical frailty together with nutritional and physiotherapeutic assessments should prompt active prehabilitation programs that were demonstrated to improve the periprocedural outcomes [6].

Physical frailty can be assessed with over 20 different screening tools. They vary in the type and number of components being assessed. Linda Fried’s physical frailty phenotype index is the one the most often used [1]. From the methodological point of view, it combines objective and subjective measures of a subject’s physical performance [1]. They both might be biased in case of a subject’s cognitive impairment, as the later may have an impact on almost all fields where the assessment is based on the information obtained from the participant.

Physical frailty has been associated with lower levels and steeper decline in cognitive functions [7–12]. The co-occurrence of frailty syndrome with impaired cognitive functions resulted in the creation of the concept of cognitive frailty defined as the simultaneous presence of physical frailty operationalized with the frailty phenotype model and cognitive impairment but not entitling to a diagnosis of dementia [13]. This coexistence of physical and cognitive deficits may be explained not only by physical interrelationships but also by common pathophysiological pathways [13, 14]. In addition, relationship between individual frailty components and cognitive impairment was found [7, 15–18] but the relation between specific frailty criteria and the impairment of the specific domains of cognition or the severity of cognitive impairment has not been studied in detail. We aimed to present the national prevalence estimates for physical frailty syndrome in the population of older community dwelling adults in Poland and assess the relationship (co-occurrence) of individual frailty components and impairment in different domains of cognition assessed with the Mini-Mental State Examination (MMSE) tool.

## Materials and methods

### Study population

The study group consisted of participants of the PolSenior2 project, a nationwide, multicenter, cross-sectional study of health and its determinants in old age conducted in Poland in 2018 to 2019 [19]. Out of 5987 community-dwelling people aged 60 and over selected in three-stage proportional sampling procedure, stratified by age and sex, 5378 participants with non-missing data were included in present analysis.

### Study procedures

The study staff received training in all procedures employed. Participants who consented to take part were examined in their home environment. The study protocol and procedures were described previously in detail, elsewhere [19].

The data concerning participants’ health status were obtained through questionnaires administered to participants and were supplemented by questioning caregivers or consulting medical records, when available.

Briefly, frailty syndrome was diagnosed based on Fried’s physical frailty phenotype criteria and comprised the assessment of weakness, slowness, self-reported exhaustion, low level of physical activity, and unintentional weight loss [20]. We used a handheld hydraulic dynamometer (Saehan SH5001) to assess handgrip strength [21]. Weakness was defined based on the cutoff values for handgrip strength as established by Fried et al. [20]. Time to walk 3 m at usual walking speed was measured; if impossible because of home environment or safety conditions, a shorter walking path in a subject’s home was used. Slowness was diagnosed in accordance with the walking time cutoffs, stratified by sex and height [20]. Exhaustion was diagnosed based on the report of a subject’s positive response to at least one of the two modified questions of the Center for Epidemiologic Studies Depression Scale (CES-D): “I felt that everything I did in the last week was an effort” and “I could not get going” [22]. In patients with marked cognitive decline (MMSE < 19), exhaustion was assessed based on the description by the caregiver. The 7-day Physical Activity Recall (PAR) scale was used to collect information on the type and duration of the subject’s physical activity in the last 7 days before the examination [23]. Based on the PAR, the individual energy expenditure per week was estimated and classified according to Fried’s low level of physical activity thresholds (with < 383 kcal/week and < 270 kcal/week for men and women respectively considered as demonstrative of frailty syndrome) [20]. We recorded weight (in kilograms) using the Tanita BC-545 N Segmental Body Composition Scale, and unintentional body weight loss was diagnosed if a loss of > 4.5 kg or ≥ 5% in the past 6 months was detected. Frailty was diagnosed as the presence of 3 or more criteria and pre-frailty as the presence of 1 or 2 criteria [20].

Participants were screened for cognitive impairment with the MMSE inventory. We used the Polish version of the MMSE, translated with the permission of Psychological Assessment Resources Inc.16204 North Florida Ave, Lutz, Florida 33,549, USA, and published by the Psychological Testing Laboratory of the Polish Psychological Association [24, 25]. Based on the MMSE score, participants were classified as suspected dementia when scoring < 24 points [26]. The MMSE cutoffs for suspected MCI, suspected mild, suspected moderate and suspected severe dementia were, respectively, 24–26, 19–23, 11–18, and 0–10. These cutoffs reflected the available literature [26] and the consensus in the PolSenior2 group [27].

The study was conducted according to the WMA Helsinki declaration and was approved by the Bioethics Committee of the Medical University of Gdansk (NKBBN/257/2017). Written informed consent to take part was obtained from the participants or their proxies.

### Statistical analyses

The data management and the statistical analyses were performed with R version 3.6.3 R (R Core Team, version 3.6.3.) and SAS 9.4 TS Level 1M5. The continuous variables were compared with t-test or Mann–Whitney U test for two groups and ANOVA or Kruskal–Wallis test in case of three or more groups, normally and non-normally distributed variables, respectively. The proportions were compared with Chi-square test. To model probability of dementia and its severity as function of frailty components, we fitted logistic regression models with adjustment for sex and age. Sampling weights were included in statistical calculations to account for the complex survey design using R survey package. The post-stratification procedure was used to match age–sex sample distribution to the population of Poland. The 2-tailed tests were carried out with significance level of p ≤ 0.05.

## Results

Mean (standard deviation—SD) age of 5378 participants (58.0% women) was 75.0 (9.4) years. In the study group, frailty according to the Fried criteria was diagnosed in 23.5% of respondents, and 54.6% were pre-frail.

The characteristics of the study group divided into subgroups of frailty status are presented in Table 1. The mean age of robust participants was lower by 4 years than of the pre-frail and by 11.3 years than that of frail individuals. Compared to the robust group, pre-frail and frail participants more often had suspected mild to severe dementia. The frail participants had lower level of education as expressed by highest percentage of persons with primary education, and the lowest proportion of those with vocational, secondary and higher education, compared to the pre-frail and robust participants, and they more often worked in blue-collar jobs or were farmers. Frailty and pre-frailty were less common among married participants and more common among widows and widowers (Table 1).

**Table 1:** Group characteristics regarding frailty status

Parameter	Robust N = 1181	Pre-frail N = 2942	Frail N = 1255	p
Age (years (95% CI))	66.7 (66.4–66.9)	70.7 (70.4–71.0)	78.0 (77.3–78.7)	< 0.001
Cognitive status % (95% CI)				< 0.001
Normal	75.3 (71.9–78.7)	59.5 (56.1–62.9)	35.3 (31–39.6)	
MCI	19.1 (16.3–22)	25.1 (22.7–27.5)	18.8 (15.8–21.8)	
Mild dementia	4.9 (3.5–6.3)	12.5 (10.6–14.3)	23.9 (20.8–27.1)	
Moderate dementia	0.6 (0.2–1.1)	2.6 (1.9–3.3)	14 (11.3–16.7)	
Severe dementia	0	0.4 (0.2–0.6)	8 (6.4–9.7)	
Smoking % (95% CI)	17.5 (13.6–21.3)	13.6 (11.8–15.3)	10.5 (7.2–13.8)	
Education (95% CI)				< 0.001
Primary	12.0 (9.1–14.9)	25.7 (21.7–29.7)	44.8 (39.2–50.4)	
Vocational	28.7 (24.5–33.0)	27.8 (25.1–30.5)	20.7 (16.9–24.5)	
Secondary	39.3 (35.4–43.2)	34.2 (31.4–37.0)	26.4 (21.9–30.9)	
Higher	20.0 (15.5–24.4)	12.3 (9.8–14.8)	8.1 (5.5–10.7)	
Residence % (95% CI)				NS
Rural	35.8 (27.0–44.6)	38.8 (31.9–45.6)	41.2 (33.2–49.2)	
Small town (< 50 thousand)	25.2 (15.9–34.6)	20.6 (15.6–25.7)	19.8 (14.0–25.6)	
Medium-sized town (50–200 thousand)	14.2 (8.5–20.0)	19.0 (14.0–24.0)	15.1 (10.3–19.9)	
Cities > 200 thousand	24.7 (11.4–38.0)	21.6 (11.0–32.2)	23.9 (12.5–35.3)	
Occupation % (95% CI)				< 0.001
Blue collar	44.1 (39.4–48.8)	50.7 (46.4–55.0)	57.2 (52.2–62.1)	
White collar	38.8 (33.6–43.9)	32.5 (28.4–36.5)	25.8 (20.7–30.8)	
Farmer	4.0 (1.8–6.2)	7.4 (4.8–10.1)	9.9 (6.7–13.2)	
Services	10.8 (8.1–13.4)	7.7 (6.4–9.1)	5.8 (3.7–7.9)	
Marital status % (95% CI)				< 0.001
Not married	3.8 (2.1–5.5)	3.1 (2.3–3.8)	5.1 (2.9–7.2)	
Married	75.4 (72.5–78.3)	63.8 (61.8–65.9)	44.5 (40.5–48.4)	
Widow/widower	15.4 (13.2–17.6)	28.5 (26.4–30.5)	47.1 (43.2–50.9)	
Divorced/separated	5.4 (3.2–7.6)	4.7 (3.6–5.7)	3.4 (1.7–5.0)	

**Fig. 1:**
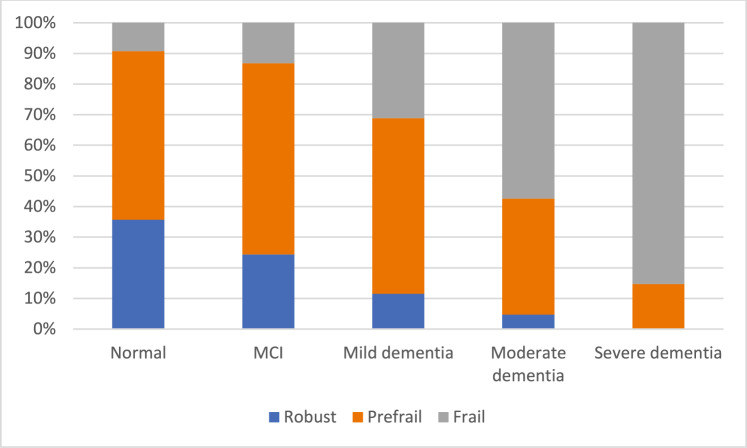
Frailty status and cognition

2,758 (51.3%) respondents had a normal result in the MMSE. Figure 1 presents the percentage distribution of frailty status depending on strata of cognitive impairment. The percentage of robust participants declined across the increasing stage of cognitive impairment from 35.7% in normal cognition group, 24.3% in suspected MCI, 11.5% in suspected mild dementia, to 4.7% in suspicion of moderate dementia and no robust individuals in the group with the MMSE range suggesting severe dementia. The highest percentage of pre-frail participants was found in MCI group (62.4%), followed by suspected mild dementia (57.3%), normal cognition (55.1%), suspicion of moderate dementia (37.9%) and suspected severe dementia (14.7%). Consequently, the percentage of persons with frailty syndrome increased from 9.2% in normal cognition group through MCI (13.2%), suspected mild dementia (31.1%), suspected moderate dementia (57.4%) to suspected severe dementia (85.3%).

In the next step, we assessed the prevalence of cognitive domains depending on the frailty status (Table 2). Compared to robust participants, the pre-frail, and frail individuals showed greater deficits in attention and short-term memory, and participants with frailty syndrome compared to the other two groups had worse orientation, language and visuo-spatial capabilities (Table 2).

**Table 2:** The MMSE-based scores of cognitive domains by frailty status and presence of frailty components. Data are median (interquartile range)

Frailty status	Cognitive domain					
	Orientation (max. 10)	Registration (max. 3)	Attention (max. 5)	Short-term memory (max. 3)	Language (max. 8)	Visuo-spatial abilities (max. 1)
Robust	10 (10–10)	3 (3–3)	5 (4–5)	3 (2–3)	8 (7–8)	1 (0–1)
Pre-frail	10 (10–10)	3 (3–3)	5 (2–5)	2 (1–3)	8 (7–8)	1 (0–1)
Frail	10 (8–10)	3 (3–3)	3 (1–5)	1 (0–3)	7 (6–8)	0 (0–1)
*Frailty components*						
Exhaustion	10 (8–10)	3 (3–3)	3 (1–5)	2 (0–3)	7 (6–8)	0 (0–1)
Slowness	10 (9–10)	3 (3–3)	4 (2–5)	2 (1–3)	8 (7–8)	1 (0–1)
Weakness	10 (9–10)	3 (3–3)	4 (1–5)	2 (1–3)	7 (6–8)	1 (0–1)
Weight loss	10 (10–10)	3 (3–3)	5 (2–5)	2 (1–3)	8 (7–8)	1 (1–1)
Low physical activity	10 (8–10)	3 (3–3)	3 (1–5)	2 (0–3)	7 (6–8)	1 (0–1)

Orientation—sum of MMSE questions 1–10; registration—sum of MMSE questions 11–13; attention—sum of MMSE questions 14–18; short-term memory—sum of MMSE questions 19–21; language—sum of MMSE questions 22–29; visuo-spatial abilities—MMSE question 30

**Fig. 2:**
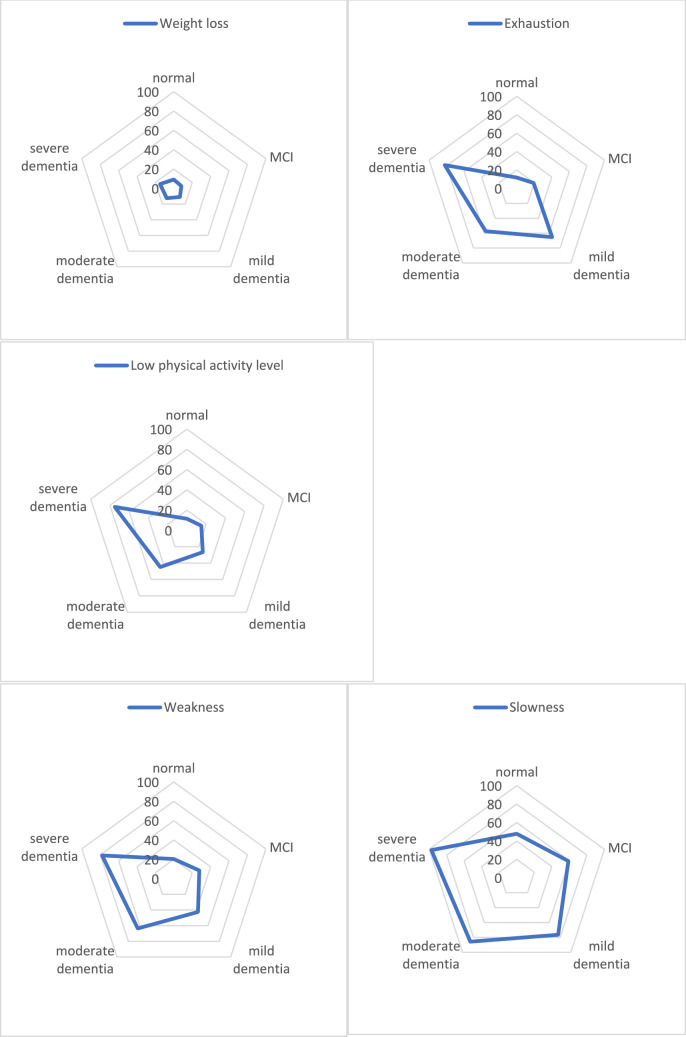
Frequency of occurrence of frailty components across strata of cognitive impairment

The analysis of prevalence of individual frailty components across the strata of cognitive impairment (Fig. 2) shows that the rarest feature in all analyzed groups was weight loss, with the highest percentage in suspected severe dementia, while the most common was slowness. People with MCI and suspected moderate dementia most frequently demonstrated slowness and weakness, whereas slowness and exhaustion were the most common in suspicion of mild and severe dementia groups. The best-preserved cognitive function in association with all individual frailty components was registration, followed by language functions and visuo-spatial abilities. The presence of each of the individual frailty components was accompanied by impaired short-term memory and attention (Table 2).

The frailty component that was associated with abnormalities in all MMSE was exhaustion. In the presence of exhaustion, the best-preserved function was registration, and the most pronounced were attention deficits. Similarly, although to a lesser extent, slowness, low muscle strength and low physical activity were associated with impairment in individual MMSE domains. Less often, deterioration of cognitive functions in the form of impaired attention, short-term memory and language functions accompanied a weight loss (Table 2).

Table 3 presents the odds of co-occurrence of cognitive impairment as a function of the presence of individual components of the frailty syndrome, after adjustment for sex and age.

**Table 3:** The odds ratios (95% CI) of cognitive impairment associated with individual frailty components, adjusted for sex and age

Frailty component	OR	95% CI		P-value
Model 1; dementia + MCI vs normal cognition				
Exhaustion	2.13	1.83	2.48	< 0.001
Slowness	1.73	1.52	1.97	< 0.001
Weakness	1.98	1.74	2.25	< 0.001
Weight loss	0.90	0.74	1.10	0.30
Low physical activity	1.45	1.24	1.69	< 0.001
Model 2; MCI vs normal cognition				
Exhaustion	1.46	1.21	1.76	< 0.001
Slowness	1.36	1.17	1.58	< 0.001
Weakness	1.52	1.30	1.78	< 0.001
Weight loss	0.88	0.69	1.12	0.297
Low physical activity	1.07	0.88	1.31	0.47
Model 3; mild dementia vs normal cognition				
Exhaustion	2.48	2.03	3.02	< 0.001
Slowness	2.27	1.86	2.76	< 0.001
Weakness	2.11	1.77	2.53	< 0.001
Weight loss	0.89	0.67	1.17	0.39
Low physical activity	1.51	1.23	1.86	< 0.001
Model 4; moderate dementia vs normal cognition				
Exhaustion	3.64	2.74	4.82	< 0.001
Slowness	3.30	2.22	4.89	< 0.001
Weakness	3.82	2.85	5.11	< 0.001
Weight loss	0.83	0.55	1.27	0.40
Low physical activity	2.49	1.87	3.33	< 0.001
Model 5; severe dementia vs normal cognition				
Exhaustion	6.05	3.54	10.34	< 0.001
Slowness	24.58	3.36	180.06	0.002
Weakness	4.15	2.25	7.66	< 0.001
Weight loss	0.38	0.15	0.95	0.04
Low physical activity	4.36	2.59	7.35	< 0.001

The probability of suspicion of dementia was greater in persons with exhaustion, slowness, weakness, and low physical activity, but not weight loss (Table 3). The probability of MCI was greater in participants with exhaustion and weakness (Table 3).

After adjustment for age and sex, the highest odds of co-occurrence of suspicion of mild and moderate dementia were greater for exhaustion, in suspected mild dementia followed by slowness and weakness, and in suspected moderate dementia by weakness and slowness. The highest odds of finding suspicion of severe dementia were greatest for slowness followed by exhaustion and low physical activity.

## Discussion

We found a positive relation between greater prevalence of physical frailty components and worse cognition assessed with the MMSE. The predominant components of frailty were slowness and exhaustion, dominating in suspected severe dementia. Persons with exhaustion predominantly presented with problems with attention and short-term memory and to a lesser degree inadequate orientation. Similar pattern was observed in persons with the diagnosis of physical frailty.

Our results, based on data from a large survey, representative at a national level, we confirmed that physical frailty and cognitive impairment tend to associate with one another. The novel finding that we present is the mapping of the association between the components of physical frailty assessed according to Fried et al. [20] and the components of cognitive impairment assessed with the MMSE [24–26].

Our observation confirms the results of other cross-sectional [7–9, 15, 28–31] and prospective studies [10, 11, 18, 32], showing a bidirectional relationship between frailty status and the presence of cognitive functions deficits and their severity. Although the interdependencies are studied and the topic exists in the literature, comparisons are difficult due to differences in methodology and research tools used. In study by Chen et al., frailty was associated with domain-specific scores for visuo-spatial abilities and attention and pre-frailty only with impaired attention [11]. Similarly, in the study by Robertson et al. (The Irish Longitudinal Study on Ageing; TILDA), frail participants scored significantly lower than the robust and the pre-frail participants in the test of global cognitive functions, memory, and attention, but the TILDA study was based on a slightly different methodology (in addition to the MMSE, they used other tests to assess memory and attention, and among the domains of cognitive functions, only memory, attention, and executive functions were assessed) [29]. In the China Comprehensive Geriatric Assessment Study, Ma et al. found that frail participants performed worse in global cognition and all ten domains than the robust and the pre-frail individuals [7], whereas in the Brazilian FIBRA Study, apart from global cognition, frailty was associated with deficits in orientation and registration [31]. In our cohort, the latter cognitive domain was the one that was best preserved among the frail participants of study. In an analysis of 10,388 participants of the Living Profiles of Older People Survey aged 65 and over, Han E et al. showed that higher scores in time orientation, registration, and attention were associated with a lower likelihood of frailty after adjusting for confounders [28].

Of the frailty components, we found that slow gait was associated with all levels of cognitive impairment. This is important finding adding to the body of evidence linking slow gait with a wide range of complications in older adults [33, 34]. The second most common criterion in participants with a suspicion of severe dementia and the most common in suspected mild and moderate dementia (after adjustment for age and sex) was exhaustion, while the third in line was weakness, which also concerned MCI. The rarest feature regardless of the cognitive impairment stage was weight loss. Previously, slowness and exhaustion were shown to be the frailty criteria that were most often associated with cognitive impairment. In a prospective National Health and Aging Trends study of 7439 older people, the most prevalent frailty components were exhaustion and low physical activity, followed by slow gait speed and weakness. Similarly, to our results, weight loss was least prevalent [17]. In a prospective evaluation, they showed that the components associated with cognitive decline were slowness, weight loss and weakness, with slow gait explaining more of the variance in cognitive function than frailty and other frailty criteria [17].

Comparable results were obtained by Ma et al. In their study, the MMSE score correlated positively with walking speed, but they did not assess muscle strength. In the logistic regression analysis after adjusting for age, gender, education level, living area and chronic conditions, they found that the level of cognitive function was associated with exhaustion, followed by slowness and inactivity, while weight loss was not associated with cognitive impairment [7]. In addition, in the study by Brigola et al., except for an unintentional weight loss, all frailty components were more prevalent in older adults with cognitive impairment than in individuals with intact cognition. In the regression analysis after adjustment for age, education, place of residence, functionality, and other frailty criteria, only exhaustion and slowness remained associated with cognitive impairment [15]. In many other studies, the relationship between cognitive function and frailty criteria was assessed, with varied results. Some studies reported slow gait as the main frailty component associated with cognitive impairment [35–37]. Robertson et al. in their systematic review found that gait speed and grip strength were the components of frailty strongly associated with overall cognitive function, while frailty status was related to executive function and attention [12]. A range of explanations have been offered for these observations, however, inconsistent. Rosano et al. have found that both slow gait and cognitive function were associated with smaller volume of the prefrontal area of the brain [38]. There is also evidence that slow gait may predict cognitive decline [39, 40], and when combined with memory disorders it constitutes the motor-cognitive risk syndrome characterized by high risk of conversion to dementia [41]. It has also been demonstrated that slow gait is most often associated with vascular dementia, with microvascular disease in the subcortical area being responsible for gait automatism [30, 42]. This concept may be supported by the results of studies in which frailty was associated with vascular cognitive disorders [43–45]. On the other hand, Buchman et al. found the association between higher levels of frailty with the accumulation of neuropathology typical of Alzheimer’s disease (AD) and suggested that frailty might be prodromal sign of the latter [16, 46].

The importance of our study rests is the analysis of the relationship between individual frailty components and various degrees of cognitive impairment and suspected dementia.

Analyzing the relationship between frailty criteria and domains of cognitive functions, we found that the best-preserved cognitive function in association with all individual frailty components was registration, while the most common cognitive dysfunction accompanying all individual components of frailty was impairment of short-term memory and attention. Attention deficits, in addition to executive functions, were also indicated as the most frequently associated frailty syndrome in the systematic review by Robertson et al. [12].

Exhaustion was the frailty component associated with the abnormalities in all MMSE domains. In the presence of exhaustion, the best-preserved function was registration, and the most affected cognitive function was attention. Other frailty criteria associated with impairment in individual MMSE domains were slowness, low muscle strength and low physical activity. Weight loss was less frequently associated with cognitive deficits, a relationship was found with impaired attention, short-term memory and language functions. The assessment of the relationship between individual frailty criteria and cognitive functions was carried out by Robertson et al. in the TILDA study; however, they used different cognitive assessment tools [29]. In contrast to our results, in their analysis, exhaustion was only associated with global cognitive functions. Among the remaining frailty components, only slow gait was associated with attention and executive functions, and weakness with global cognition and executive functions [29]. Another study, by Lin et al. which also used cognitive assessment tools other than ours, found a relationship between slowness and memory, attention and language functions [30]. But out of frailty criteria, they evaluated only speed gait and grip strength.

The explanation for the association between frailty and cognitive impairment may be the common pathogenetic pathways of frailty and neurodegenerative disorders, which include neuropathological changes, chronic inflammation, endocrine dysregulation, accumulation of DNA damage, mitochondrial dysfunction, and oxidative stress resulting in protein and lipid damage [12–14].

The relationship between these pathologies is bidirectional. Several cross-sectional and longitudinal studies have shown that frail people are more likely to have cognitive impairment or dementia and those who already have dementia are more likely to be frail [12]. Among others, multivariate regression in the study by Ma et al. revealed that participants with frailty syndrome had 2.5-fold increase in the risk of cognitive impairment after adjusting for age, gender, education level, living area, and chronic diseases [7]. In addition, Chen et al. showed that frail persons were over two times more likely to experience cognitive decline compared to non-frail persons [10].

The opposite direction of the relationship was assessed by Han et al. [28], and Chen et al. [11]. It has been shown that cognitive impairment measured with MMSE was associated with increased risk of frailty by over 80% in men and nearly 70% in women after controlling for all covariates [28].

The coexistence of frailty and cognitive disorders has serious consequences as increased risk of disability, loss of independence, increased morbidity and severity of disease, poor quality of life, risk of hospitalization, institutionalization and a greater risk of death [47, 48]. It is suggested that physical frailty is a stronger indicator of functional limitation and global cognition than age [49]. Feng et al. established that frail older adults with cognitive impairment had fivefold increase in mortality risk, 12-fold increase in functional disability, and lower quality of life compered to people with frailty or cognitive impairment alone [50].

Our results might have potential implications for public health. Persons affected with both physical frailty and cognitive impairment are those who are in need of assessment, counseling and rehabilitation and dietary intervention. On the other hand, our cross-sectional study was not designed to demonstrate effectiveness of possible intervention directed on prevention of frailty and cognitive decline. However, implementation of rehabilitation and appropriate dietary interventions has been demonstrated to be of value both in prevention and, as an adjunct, in therapy of both frailty and cognitive impairment. However, more studies are needed before more specific recommendations could be ultimately formulated.

Our results need to be considered in the context of the study limitations. First, our study was cross-sectional and thus causality cannot be established. Second, the cognitive assessment was performed only with a screening tool, and we could only suspect dementia. We did not have the possibility to address the precise type of dementia, and various types of dementia could display different patterns of association with frailty and its components. For the presented analyses, we used the uncorrected MMSE results. This stems from the fact that the currently accepted corrections have not been based on the Polish population data and might thus yield biased results. Second, the data we present are estimates at the national level, and they were derived with use of the preplanned sampling weights. Use of age and education adjusted MMSE values might cause bias. Finally, in the age-adjusted analyses, we might face the problem of overadjustment.

The major strength of our study rests on the fact that it was designed to be representative of the population of older persons in Poland, and the results are thus generalizable at the national level.

In conclusion, frailty and cognitive impairment are closely associated; however, both individual frailty components and the individual cognitive domains contribute differently to this relation. While exhaustion was standing out among the frailty components, it was associated with impaired orientation and attention deficits. Our data, especially after independent confirmation, may inform both the preventative and therapeutic actions, especially in the realm of rehabilitation and nutritional counseling. 

## Data Availability

Upon reasonable request data would be made available pending the decision by the study managing committee.
